# Current and Emerging Energy Sources for Atrial Fibrillation Ablation: A Comparative Analysis of Clinical Efficacy, Safety, and Procedural Implementation

**DOI:** 10.3390/jcm15020751

**Published:** 2026-01-16

**Authors:** Cristian Martignani, Giulia Massaro, Alberto Spadotto, Jennifer Oppimitti, Maria Carelli, Andrea Angeletti, Alessandro Carecci, Igor Diemberger, Mauro Biffi

**Affiliations:** 1 Cardiology Unit, IRCCS Azienda Ospedaliera-Universitaria di Bologna, 40138 Bologna, Italy; 2 Department of Medical and Surgical Sciences, Institute of Cardiology, University of Bologna, Via Massarenti 9, 40138 Bologna, Italy; 3 Pharmacy Unit, IRCCS Azienda Ospedaliera-Universitaria di Bologna, 40138 Bologna, Italy

**Keywords:** atrial fibrillation, catheter ablation, radiofrequency ablation, cryoballoon, pulsed-field ablation, irreversible electroporation, nanosecond PFA, health economics, cost-effectiveness

## Abstract

Atrial fibrillation (AF) management has historically relied on thermal ablation modalities—radiofrequency (RF) and cryoballoon—which have established a high benchmark for pulmonary vein isolation (PVI). However, the inherent risk of collateral thermal injury and lesion inconsistency has driven the search for alternative energy sources. The recent clinical adoption of pulsed-field ablation (PFA), based on irreversible electroporation, represents a significant technological evolution. This narrative review provides a critical appraisal of the transition from thermal to pulsed-field technologies. We synthesized data from pivotal trials and recent health-economic analyses to evaluate the biophysical mechanisms, clinical efficacy, and safety profiles of contemporary devices. We conduct a head-to-head comparison of all modalities regarding critical safety endpoints (esophageal, neurological, and vascular), real-world procedural challenges (anesthesia, lesion assessment), and economic sustainability. While PFA offers distinct advantages in procedural speed and tissue selectivity, we highlight that thermal modalities—particularly cryoballoon and very-high-power RF—retain competitive profiles in terms of cost-effectiveness and established long-term durability. This review aims to provide a balanced roadmap for clinicians navigating the complex choice between established thermal efficacy and the promising, yet evolving, landscape of electroporation.

## 1. Introduction

Atrial fibrillation (AF) represents a rapidly growing global health burden. Epidemiological data project a doubling of AF prevalence by 2050, driven by an aging population and improved survival from comorbid conditions [1]. Beyond its profound impact on quality of life, AF is independently associated with a 1.5- to 3.5-fold increase in mortality and a significant risk of stroke, heart failure, and cognitive decline [2]. Consequently, the therapeutic paradigm has shifted from palliative rate control to early and aggressive rhythm control strategies, aiming to modify the natural history of the disease [3].

Catheter ablation has progressively emerged as the cornerstone of this strategy. Initially reserved for drug-refractory symptomatic patients, current guidelines and compelling evidence from trials such as EARLY-AF and STOP-AF First now position ablation as a viable first-line option for paroxysmal AF [2,4,5]. Central to this therapeutic success is the concept of pulmonary vein isolation (PVI), based on the fundamental recognition that ectopic beats originating from muscular sleeves within the pulmonary veins (PVs) trigger the majority of AF episodes [6].

For over two decades, the achievement of PVI has relied predominantly on thermal injury—either heating tissue through radiofrequency (RF) energy or freezing it via cryothermal systems. These modalities have matured significantly, establishing a high benchmark for efficacy with 1-year success rates hovering between 70% and 80% for paroxysmal AF. However, thermal ablation carries intrinsic biophysical limitations. The indiscriminate nature of thermal propagation results in a persistent risk of collateral damage to critical non-target structures, such as the esophagus (atrio-esophageal fistula), the phrenic nerve, and the pulmonary vein ostia (stenosis) [7,8]. Furthermore, the reliance on conductive heat transfer can lead to incomplete lesion formation in areas of thick tissue or heat-sink effect, contributing to late arrhythmia recurrence.

The past decade has shown an accelerated shift toward non-thermal modalities to overcome these safety and durability barriers. Pulsed-field ablation (PFA), based on the principle of irreversible electroporation (IRE), has generated significant clinical interest. By delivering high-voltage electrical pulses that destabilize cell membranes, PFA offers the theoretical advantage of tissue selectivity—sparing the extracellular matrix and adjacent vascular or neural structures [9].

**Figure 1:**
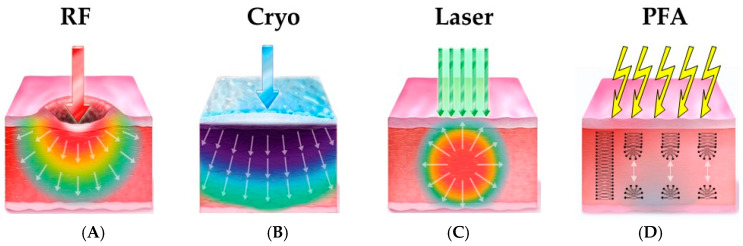
Biophysical mechanisms of lesion formation across ablation modalities. (**A**) Radiofrequency (RF): Lesions are created via resistive heating at the catheter tip and passive conductive heating into deeper tissue, causing coagulative necrosis. Note the potential for thermal spread to collateral structures. (**B**) Cryoballoon: Utilizes the Joule-Thomson effect to freeze tissue, causing intracellular ice crystal formation and microvascular stasis (freeze-thaw injury). Efficacy relies on balloon occlusion. (**C**) Laser Balloon: Delivers photothermal energy (980-nm diode) directed visually. Light is absorbed by tissue water, converting to heat. (**D**) Pulsed-Field Ablation (PFA): Uses high-voltage microsecond or nanosecond pulses to create an electric field. This causes dielectric breakdown of the cell membrane, forming permanent nanopores (irreversible electroporation). The mechanism is non-thermal and tissue-selective, sparing the extracellular matrix and adjacent nerves.

As highlighted in comprehensive frameworks by Brasca et al., the landscape of AF ablation is no longer a binary choice but a spectrum of evolving technologies [10]. This narrative review provides an extensive and critical analysis of both thermal and non-thermal ablation energies (Figure 1). We dissect the biophysical underpinnings, scrutinize the latest clinical evidence from randomized trials and large registries, and evaluate the economic sustainability of these innovations. Particular attention is devoted to emerging PFA technologies—including circular loop systems, balloon-guided platforms, and nanosecond PFA—assessing their potential to redefine the standard of care in electrophysiology.

## 2. Methods

This narrative review is based on a comprehensive literature search performed on PubMed and Embase. The search strategy employed combinations of keywords: (‘Atrial Fibrillation’ OR ‘AF’) AND (‘Ablation’ OR ‘PVI’) AND (‘Pulsed Field’ OR ‘Radiofrequency’ OR ‘Cryoballoon’ OR ‘Laser’). The search primarily focused on articles published between January 2015 and February 2025 to reflect contemporary technological progress; however, selected landmark studies (e.g., Haïssaguerre et al., 1998 [6]) and fundamental biophysical papers published prior to this window were intentionally included to provide essential historical and mechanistic context. Out of 452 records identified and screened for relevance, 41 articles were ultimately included. Selection criteria focused on randomized controlled trials, large-scale registries, and health-economic analyses, while modalities not in mainstream clinical use (e.g., microwave, high-intensity focused ultrasound) were excluded.

## 3. Thermal Modalities: Established Standards and Persistent Limitations

Thermal ablation remains the standard of care against which all new technologies must be measured. Understanding the specific mechanisms and limitations of these established modalities is essential to contextualize the rise in PFA (Table 1).

### 3.1. Radiofrequency and the Pursuit of Lesion Durability

Radiofrequency (RF) ablation relies on resistive heating at the electrode-tissue interface, followed by conductive heat transfer to deeper myocardial layers [11]. The durability of PVI with RF has historically been hampered by catheter instability and variable tissue contact. The introduction of contact force-sensing catheters represented a major leap forward, allowing operators to titrate energy delivery based on real-time feedback [12].

The most recent evolution in this field is Very-High-Power Short-Duration (vHPSD) RF (e.g., 90 W for 4 s). This strategy aims to shift the lesion formation mechanism almost entirely to resistive heating, creating wider but shallower lesions. The theoretical benefit is improved catheter stability (shorter application time reduces movement artifact) and reduced conductive heating to collateral organs. Clinical data confirm that vHPSD is non-inferior to standard RF in safety and efficacy, with significantly reduced procedural times [13,14]. However, intrinsic limitations remain: the risk of “steam pops” (explosive tissue vaporization) persists if irrigation is inadequate, and achieving transmurality in thick ventricular or ridge tissues without conductive heating remains challenging.

### 3.2. Cryoballoon: Efficiency and Anatomical Considerations

Cryoballoon ablation standardized PVI by reducing operator dependency. By utilizing the Joule-Thomson effect to freeze tissue to −40 °C/−60 °C, the cryoballoon creates a circumferential lesion in a single application. The landmark FIRE AND ICE trial demonstrated its non-inferiority to RF, establishing it as a primary tool for paroxysmal AF [15,16].

The main limitation of the cryoballoon is its dependence on anatomy. Effective heat transfer requires a perfect seal (occlusion) of the vein. In patients with common ostia, large veins, or oval anatomies, achieving occlusion can be difficult, leading to gaps or the need for “pull-down” maneuvers that may compromise safety. Furthermore, the proximity to the phrenic nerve during right-sided ablation requires continuous pacing and monitoring, with a residual risk of transient or persistent palsy [17].

### 3.3. Laser Balloon: Visual Precision and Versatility

Laser balloon technology offers a distinct approach by combining a compliant balloon with a rotatable laser arc under direct endoscopic visualization. This allows the operator to see the tissue contact and titrate energy delivery point-by-point within the balloon, overcoming the “occlusion dependency” of the cryoballoon [18].

While often considered a niche technology due to a steeper learning curve, recent data reinforce its relevance. A large international multicenter study by Martignani et al. analyzed outcomes in a real-world cohort, demonstrating that laser balloon ablation achieves high rates of durable isolation with a favorable safety profile. The study highlighted the device’s utility in complex anatomies where fixed-shape balloons fail, suggesting that for experienced centers, the laser balloon remains a highly precise thermal alternative [19].

## 4. Biophysics of Electroporation: Beyond the “Non-Thermal” Label

To understand the clinical behavior of PFA, one must look beyond the simplified label of “non-thermal energy”. PFA applies ultra-short, high-voltage electrical fields (typically 1000–2000 V/cm) that induce a transmembrane potential difference. When this difference exceeds a critical threshold, hydrophilic pores form in the phospholipid bilayer (electroporation). If the field strength is sufficient, these pores become permanent, leading to loss of homeostasis and apoptotic cell death (irreversible electroporation, IRE) [20].

**Figure 2:**
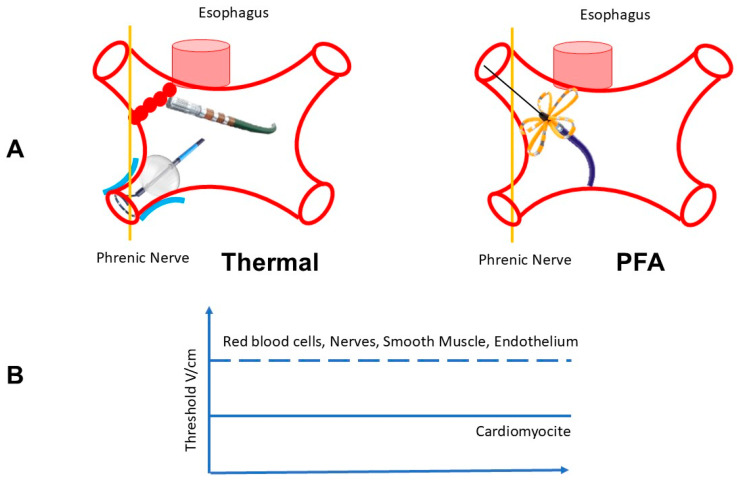
Tissue selectivity and the concept of the therapeutic window. (**A**) Anatomical Comparison: Cross-sectional view of the posterior left atrial wall and adjacent esophagus. Left (Thermal): Radiofrequency or Cryo energy propagates indiscriminately through tissue conductivity. If the lesion extends beyond the myocardium, it causes collateral thermal injury to the esophageal wall (risk of atrio-esophageal fistula). Right (PFA): The electric field penetrates both tissues. However, while cardiomyocytes undergo irreversible electroporation (necrosis), the esophageal smooth muscle and nerve axons possess a higher threshold for damage, remaining structurally intact despite exposure to the field. (**B**) The Therapeutic Window: Graphic representation of cell susceptibility to electric fields. Cardiomyocytes (solid line) are larger and more sensitive, undergoing cell death at lower field strengths (e.g., 400–1000 V/cm). Collateral tissues like nerves and blood vessels (dashed line) require significantly higher field strengths to suffer irreversible damage. The operational range of clinical PFA devices targets this “Therapeutic Window”, ensuring myocardial ablation while sparing collateral structures.

The therapeutic rationale of PFA lies in tissue selectivity. The threshold for IRE depends on cell size, geometry, and orientation relative to the field. Cardiomyocytes, being large and elongated, have a lower threshold for IRE compared to the smaller fibers of the esophagus, the myelinated axons of the phrenic nerve, or the collagen matrix of blood vessels [21]. This creates a wide therapeutic window where myocardial necrosis can be achieved without damaging adjacent structures (Figure 2).

However, PFA is not entirely “non-thermal”. The high current density generates Joule heating. If pulses are not properly spaced or if the electrode is embedded in tissue without cooling, thermal effects can occur. Modern PFA waveforms (biphasic, pulse trains) are engineered to minimize this thermal component and prevent muscle capture, but the bio-physical reality is a complex interplay of electrical field distribution and thermal management.

## 5. Pulsed-Field Ablation: Technological Approaches and Critical Appraisal

**Figure 3:**
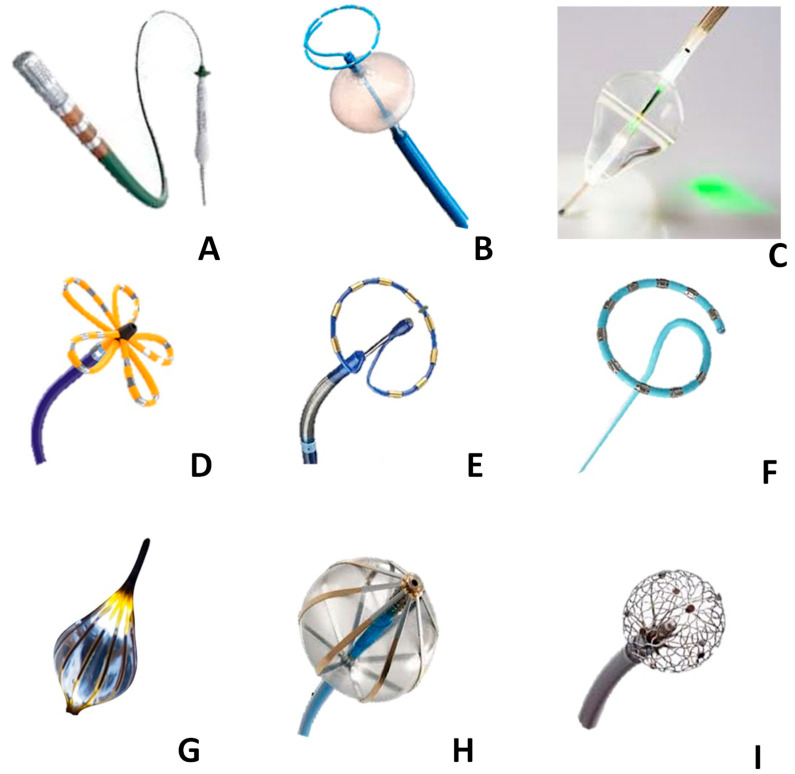
Comprehensive overview of ablation catheter technologies. (**A**) Radiofrequency (RF) Catheter: Standard point-by-point irrigated catheter equipped with contact force-sensing technology for titrated energy delivery. (**B**) Cryoballoon: Balloon-based platform designed for circumferential pulmonary vein isolation using cryothermal energy (Joule-Thomson effect). (**C**) Laser Balloon: Visually guided compliant balloon featuring a rotatable laser arc for point-by-point thermal ablation under direct endoscopic visualization. (**D**) Pentaspline PFA Catheter (e.g., Farawave): Shown in the “flower” configuration deployed for antral isolation; it can also be retracted into a “basket” for ostial stability. (**E**) Fixed Circular PFA Loop (e.g., PulseSelect): A multi-electrode loop on a tilted shaft designed for “single-shot” pulsed-field delivery. (**F**) Variable PFA Loop (e.g., Varipulse): An adjustable-diameter loop integrated with 3D electro-anatomical mapping systems for real-world lesion tagging. (**G**) Visually Guided Compliant PFA Balloon (e.g., OptiShot): Features electrodes on the balloon surface and an endoscopic camera for direct verification of tissue contact. (**H**) Balloon-in-Basket PFA (e.g., Volt): Combines an inner occlusion balloon for blood flow insulation with outer splines for targeted energy delivery. (**I**) Focal Lattice-Tip (e.g., Sphere-9): A compressible mesh-tip catheter capable of delivering both PFA and RF for versatile point-by-point ablation.

PFA is not a monolithic entity; clinical outcomes depend heavily on catheter geometry and waveform engineering. The current ecosystem can be categorized into four main design philosophies (Figure 3 and Table 2).

### 5.1. Pentaspline Catheters (Farawave)

The pentaspline design (Farawave, Boston Scientific) has spearheaded PFA adoption. It can transform between a basket (for ostial stability) and a flower (for antral coverage) configuration. The ADVENT trial, a randomized comparison against thermal standard-of-care, met its primary endpoints, showing a 73.3% success rate for PFA versus 71.3% for thermal ablation [22]. Crucially, the PFA arm had significantly less PV stenosis and zero esophageal fistulas. Beyond clinical trials, large-scale real-world registries such as MANIFEST-PF, encompassing over 1700 patients, have confirmed the high safety profile of PFA in clinical practice, reporting a major complication rate of 1.6%, which is favorably comparable to established thermal modalities [23].

- Critical Appraisal: The main limitation of the first-generation system was the lack of integration with electroanatomical mapping (“blind” procedure), necessitating heavy fluoroscopy use. While the “single-shot” approach is fast, it lacks the feedback of contact force, relying entirely on catheter positioning to ensure field coverage.

### 5.2. Circular Loop Catheters (PulseSelect, Varipulse)

These devices integrate PFA into familiar circular mapping geometries, appealing to operators accustomed to RF workflows.

- PulseSelect (Medtronic): This fixed-diameter loop was evaluated in the PULSED AF trial, achieving 66% freedom from arrhythmia at one year [20]. While safety was exceptional (0% esophageal/phrenic injury), the efficacy was slightly lower than historical thermal benchmarks, likely due to the learning curve and the fixed loop size not fitting all anatomies.
- Varipulse (Biosense Webster): This variable-loop catheter integrates fully with the CARTO 3 system. The INSPIRE trial reported 100% acute isolation and zero primary adverse events [24]. The integration with 3D mapping allows for “lesion tagging”, addressing the “blindness” of earlier systems and potentially reducing gap formation.

### 5.3. Balloon-Based Systems (Volt, OptiShot)

Combining balloon stability with PFA energy aims to ensure optimal contact and blood displacement (blood shunts electrical current, reducing efficacy).

- Volt (Abbott): Uses a “balloon-in-basket” design to insulate the blood pool while splines deliver energy [25].
- OptiShot (Galvanize): Incorporates the direct endoscopic visualization of laser balloons. This addresses a critical PFA challenge: verifying tissue contact. Visual confirmation of blood displacement could theoretically improve lesion transmurality assurance [26].

### 5.4. Focal/Lattice-Tip Catheters (Sphere-9)

The Sphere-9 (Affera/Medtronic) features a lattice tip capable of delivering both PFA and RF. The SPHERE Per-AF trial indicated high efficacy in persistent AF [27].

- Critical Appraisal: This device provides maximum flexibility for treating non-PV triggers (e.g., mitral lines, localized reentry) where single-shot tools fail. However, point-by-point PFA is slower than single-shot approaches, negating the “speed” advantage of PFA, but offering a comprehensive tool for complex substrates.

## 6. Nanosecond PFA: Theoretical Advantages vs. Clinical Reality

Current clinical PFA systems operate in the microsecond range. Nanosecond PFA (nsPFA) utilizes pulses of significantly shorter duration (e.g., 200–600 ns). The theoretical benefit rests on the chronaxie principle: pulses are too short to recruit skeletal muscle, potentially eliminating the vigorous muscle contraction seen with microsecond PFA [28]. Furthermore, shorter pulses may reduce the electrolysis responsible for microbubble formation. While promising, nsPFA is currently in the preclinical and early feasibility stage. It remains to be seen if the reduced muscle capture translates to equivalent lesion durability in large-scale human cohorts compared to more aggressive microsecond protocols [29].

## 7. Safety Profile: A Multi-Energy Comparative Analysis

Safety in AF ablation is an inevitable trade-off. No single energy source is entirely devoid of risk; rather, the risk profile shifts to different organ systems depending on the physical modality used.

### 7.1. Collateral Damage: Esophagus and Phrenic Nerve

Thermal energy sources carry the highest historical burden of collateral damage due to indiscriminate heat or cold propagation. Radiofrequency ablation is associated with a low but potentially lethal risk of atrio-esophageal fistula (0.02–0.11%), a complication that remains a primary concern despite esophageal temperature monitoring. Similarly, cryoballoon ablation is associated with phrenic nerve palsy in 2–4% of cases, necessitating continuous diaphragmatic pacing during right-sided procedures [17].

Conversely, PFA demonstrates a distinct safety advantage in this domain. Large-scale registries such as MANIFEST-PF report a near-zero incidence of esophageal fistulas and persistent phrenic palsy [23]. This clinical reality confirms the biophysical “tissue selectivity” of electroporation, which spares the collagen-rich esophageal wall and myelinated nerve axons. However, operators must remain vigilant, as transient phrenic stunning can still occur if the electric field is applied directly to the nerve.

### 7.2. Neurological Safety: Thromboembolism and Microbubbles

Cerebral embolic risk is a shared concern across all modalities, although the underlying mechanisms differ significantly. For RF and Laser ablation, the risk primarily originates from char formation (denatured protein) or thromboembolism related to long sheath times. Cryoballoon ablation carries a specific, albeit rare, risk of air embolism during balloon insertion or exchange [30].

PFA introduces a novel mechanism: the generation of microbubbles via electrolysis and thermal fluid expansion. Early investigations raised concerns regarding high rates of “silent cerebral lesions” (SCL) on MRI. However, recent data utilizing optimized waveforms suggest that SCL rates (3–10%) are comparable to those observed with thermal ablation [31]. Consequently, no modality can currently be declared “neurologically risk-free.” Emerging nanosecond PFA (nsPFA) technologies may theoretically reduce bubble formation, but definitive clinical validation is pending.

### 7.3. Vascular Complications: Stenosis vs. Spasm

The vascular risk profile also varies by energy type. Thermal modalities (RF and Cryo) are historically associated with pulmonary vein stenosis caused by inflammation and collagen retraction at the ostium. While rare in modern practice, it remains a structural risk if ablation is performed too deeply.

Conversely, PFA spares the collagen matrix, significantly reducing the risk of structural stenosis [32]. However, it introduces a functional complication: coronary vasospasm. Severe spasm has been reported during mitral isthmus ablation due to smooth muscle stimulation or receptor activation [33]. Unlike the permanent structural damage of stenosis, PFA-induced spasm is typically reversible with nitrates, yet it represents a new clinical entity requiring specific pharmacological protocols and vigilance during linear ablation.

### 7.4. Mechanical Complications: Pericardial Effusion

Regardless of the energy source, mechanical complications such as cardiac tamponade remain a persistent risk (typically 1–2%) related to transseptal access and catheter manipulation. While PFA may reduce the risk of ‘steam pops’ compared to RF, the requirement for larger-diameter sheaths and the learning curve for new catheter geometries necessitate continued vigilance regarding mechanical cardiac injury.

## 8. Real-World Challenges: The Operator’s Perspective

Beyond the controlled environment of pivotal trials, the daily clinical implementation reveals distinct operational challenges that differentiate the energy sources. Procedural efficiency, safety considerations, economic implications, and real-world implementation challenges across AF ablation modalities are synthesised in Table 3 and Table 4.

### 8.1. Workflow and Anesthesia

Regarding workflow logistics, thermal modalities currently offer advantages in simplicity. RF and Cryoballoon procedures are routinely performed under conscious sedation in many high-volume centers, facilitating rapid turnover. In contrast, current microsecond PFA systems induce significant skeletal muscle contraction. To ensure catheter stability and patient comfort, many institutions mandate General Anesthesia (GA) with deep neuromuscular blockade. This requirement can negate the procedural speed advantage of PFA, as GA induction and emergence significantly add to the total room time. While nanosecond PFA aims to allow sedation-only workflows, for the present moment, thermal modalities often provide a more streamlined anesthetic pathway [34,35]. However, anesthesia protocols are evolving; some high-volume centers are moving toward conscious sedation for PFA by using modified pulse protocols, suggesting that GA may not be a permanent constraint but rather a center-specific preference that is currently evolving.

### 8.2. Lesion Quality Assessment

A similar divergence is observed in lesion quality assessment. RF ablation benefits from decades of validation, with metrics such as Contact Force (CF) and Ablation Index/LSI providing reliable surrogates for lesion durability [12]. Cryoballoon operators utilize time-to-isolation (TTI) and balloon nadir temperature as robust predictors of success [36].

PFA, however, currently suffers from a lack of direct feedback. There is no validated metric for lesion depth or durability; success relies heavily on catheter proximity and electrogram diminution. In areas of thick tissue, such as the left atrial roof or lateral ridge, the lack of “contact force” feedback means an operator may not know if a lesion is transmural until recurrence occurs. This “blindness” represents a significant gap in the current PFA workflow compared to the data-rich environment of modern RF mapping.

### 8.3. The “Learning Curve” Factor

The learning curve also varies substantially. Laser balloon ablation has historically presented a steep learning curve due to the manual manipulation required for the laser arc. RF ablation requires moderate skill development regarding catheter stability and point-by-point mapping. Conversely, both Cryoballoon and PFA share the advantage of being “single-shot” technologies with relatively short learning curves. However, PFA requires specific training regarding catheter manipulation (e.g., basket/flower transitions) to avoid air ingress or mechanical trauma, a skill set distinct from the passive inflation of a cryoballoon [37,38].

**Table 3:** Procedural Efficiency, Safety, and Economic Comparison of AF Ablation Modalities.

Aspect	RF (Standard)	Cryoballoon	PFA (Microsecond)	nsPFA (Nanosecond, Emerging)	Notes/Evidence
Average Procedural Time	90–120 min	60–90 min	45–60 min	<45 min (preclinical/estimated)	PFA reduces “skin-to-skin” time, improving lab throughput [39]
Workflow Complexity	High point-by-point, operator dependent	Medium single-shot, vein occlusion required	Low reproducible single-shot isolation	Very low potential sedation-only workflow	Lower complexity allows higher daily case volumes
Device Cost (per procedure)	Low-Medium	Medium	High	High (next-gen)	PFA devices cost more, but reduce downstream costs [40,41]
Redo/Retreatment Rate	10–20%	10–15%	5–10%	Estimated < 5%	Durable lesions reduce need for repeat procedures [39,41]
Major Complications	AEF, PV stenosis, char (1–2%)	Phrenic nerve palsy (2–4%), PV stenosis	Rare-minimal AEF, PV stenosis	Expected lower than microsecond PFA	PFA’s tissue selectivity reduces collateral injury [22,23]
Microbubble/Embolic Risk	Moderate	Low	Low (3–10%)	Minimal	nsPFA may reduce electrolysis-related microbubbles [28]
Total Cost of Care	Medium-High	Medium	Competitive	Competitive	Includes hospital stay, anesthesia, management of complications
Economic Advantage	Limited	Moderate	High–faster workflow	High-additional savings with sedation	ICER within willingness-to-pay thresholds for PFA [39]

**Table 4:** Comparative Real-World Challenges.

Challenge	Thermal Modalities (RF/Cryo/Laser)	Pulsed-Field Ablation (PFA)
Anesthesia	Often feasible with Conscious Sedation.	Often requires General Anesthesia (muscle capture).
Lesion Feedback	Good: Contact Force (RF), Temperature/Time (Cryo), Visual (Laser).	Poor: No direct metric for depth; relies on electrogram reduction.
Anatomy	Cryo/Laser: Limited by vein size/shape (occlusion). RF: Highly adaptable.	Single-shot PFA: Limited by spline/loop contact. Focal PFA: Adaptable.
Vascular	Risk of PV Stenosis (structural damage).	Risk of Coronary Spasm (functional effect).
Learning Curve	RF/Laser: Steep/Moderate.	PFA: Short (comparable to Cryo).

## 9. Economic Analysis: The Value Equation

As healthcare systems face increasing financial pressure, the choice of energy source is often dictated by health economics, specifically the trade-off between device cost and procedural efficiency.

### 9.1. Direct Costs vs. Efficiency

From a direct cost perspective, thermal catheters (RF and Cryo) have become commodities with stable or declining prices. However, these procedures typically require longer lab occupancy (90–120 min). PFA catheters command a significant price premium, which manufacturers justify through the “efficiency dividend”-the ability to complete procedures in under 60 min. Specifically, while PFA catheters often command a price 2–3 times higher than standard RF tools, recent analyses suggest the Incremental Cost-Effectiveness Ratio (ICER) remains favorable if procedural throughput increases by 20–30%.

Recent analyses by Neužil et al. (2025) support the cost-effectiveness of PFA in high-volume, efficiency-driven systems where time saved translates directly into additional reimbursable cases [39]. However, Soleimani et al. (2025) [40] provide a crucial counterpoint: in fixed-budget systems (common in many European public hospitals), a shorter procedure does not automatically save money. If the lab cannot perform an extra case due to staffing or bed shortages, the higher upfront cost of the PFA catheter represents a net financial loss compared to thermal options.

### 9.2. Long-Term Value: The Cost of Recurrence

The ultimate economic driver in AF management is the rate of redo procedures. With recurrence rates for paroxysmal AF remaining between 15–25% for thermal modalities, the long-term value of PFA hinges on its durability. If PFA can consistently demonstrate superior long-term freedom from arrhythmia, as suggested by the ADVENT economic analysis, the reduction in costly redo procedures would justify the higher initial investment (lower Incremental Cost-Effectiveness Ratio) [41]. Consequently, economic viability is highly context dependent: high-volume centers may leverage PFA’s speed for profit, while cost-constrained centers may find optimized thermal ablation (e.g., vHPSD RF or Cryo) to be the more sustainable standard.

## 10. Conclusions

The landscape of AF ablation has evolved into a multi-polar energy ecosystem. Radiofrequency remains the most versatile tool for complex substrates, enhanced by vHPSD technology. Cryoballoon and Laser Balloon remain robust, efficient, and cost-effective standards for pulmonary vein isolation with massive longitudinal data. Pulsed-Field Ablation represents a transformative leap in safety, minimizing collateral risks to the esophagus and nerves, but introduces new challenges regarding anesthesia, visualization, and cost.

The “best” energy source does not exist in isolation. The future of electrophysiology lies in a tailored approach: utilizing PFA for speed and safety in straightforward anatomies, while retaining thermal modalities for cost-containment or complex substrate modification where feedback metrics (like contact force) are paramount.

## Figures and Tables

**Table 1:** Biophysical and Safety Comparison of Ablation Modalities.

Modality	Energy Mechanism	Target Selectivity	Muscle Capture	Microbubbles	Esophageal Risk	Hemolysis Risk	Coronary Spasm Risk
RF (Standard)	Resistive + Conductive Heating	Low	No	Low	Moderate	Low	Low
vHPSD RF	Predominantly Resistive Heating	Moderate	No	Low	Low-Moderate	Low	Low
Cryoballoon	Freezing (Joule-Thomson)	Low	No	None	Low	Low	Low
Laser Balloon	Photothermal Heating	Low	No	Low	Low-Moderate	Low	Low
PFA (Microsecond)	Irreversible Electroporation	High (Myocardium)	Yes (GA req)	High	Minimal	High	Moderate
nsPFA	Nanosecond Electroporation	High (Myocardium)	No (Sedation possible)	Minimal	Minimal	Low	Low

**Table 2:** Overview of PFA Catheter Geometries.

Geometry	Device	Mapping Integration	Key Strength	Critical Limitation
Pentaspline	Farawave	No (Gen 1)/Yes (Gen 2)	Extensive safety data (ADVENT)	“Blind” procedure (Gen 1); fluoroscopy dependent
Circular Loop	PulseSelect	No	Familiar shape for RF operators	Fixed diameter may not fit all ostia; lower efficacy in first gen
Variable Loop	Varipulse	Yes (CARTO 3)	Visualized tissue proximity	Mechanics of variable loop can be complex
Compliant Balloon	OptiShot	N/A (Visual)	Direct Endoscopic View	Learning curve for visual interpretation
Balloon-in-Basket	Volt	Yes (EnSite)	Insulation of blood pool	Virtual visualization only (no direct view)
Focal Lattice	Sphere-9	Yes (Mapping)	Flexibility (Linear/Point)	Slower (point-by-point); requires high skill

## Data Availability

No new data were created or analyzed in this study.
